# Image guided intensity modulated hypofractionated radiotherapy in high-risk prostate cancer patients treated four or five times per week: analysis of toxicity and preliminary results

**DOI:** 10.1186/1748-717X-9-214

**Published:** 2014-09-26

**Authors:** Maurizio Valeriani, Alessia Carnevale, Mattia Falchetto Osti, Vitaliana DE Sanctis, Linda Agolli, Riccardo Maurizi Enrici

**Affiliations:** Department of Radiation Oncology, La Sapienza” University, Sant’Andrea Hospital of Rome, Rome, Italy

**Keywords:** Hypofractionation, High risk prostate cancer, IMRT-SIB

## Abstract

**Background:**

To evaluate efficacy and toxicity of hypofractionated intensity-modulated simultaneous integrated boost (IMRT-SIB) and image-guided (IGRT) radiotherapy in the treatment of high-risk prostate cancer patients.

**Methods:**

Eighty-two patients with high-risk prostate cancer were analysed. An IMRT treatment was planned delivering 68.75 Gy to the prostate, 55 Gy to the seminal vesicles and positive nodes and 45 Gy to the pelvis in 25 fractions. The first 59 patients received 4 weekly fractions whereas the last 23 patients received 5 weekly fractions. All patients were submitted to hormonal therapy.

**Results:**

The median follow-up was 31 months. Acute grade 1–2 gastrointestinal (GI) toxicity rates were 13.4%. Grade 1–2 and grade 3 genitourinary (GU) toxicity rates were 22% and 1.2%, respectively.

Grade 1 and 2 GI late toxicity rates were 1.2%. No grade ≥3 toxicity was recorded. Grade 1 GU late toxicity rate was 2.4%. No grade ≥2 toxicity was recorded.

No significant difference was calculated in terms of acute and late toxicity between the group treated 4 or 5 times weekly.

The actuarial 3-years Overall survival and Freedom from biochemical failure were 98.6% and 91.3%, respectively.

**Conclusions:**

The present study demonstrated that hypofractionated IGRT-IMRT-SIB in patients with high-risk prostate cancer is efficient with acceptable toxicity profile. Outcome in terms of survival are promising, but longer follow-up is needed.

## Background

High-risk prostate cancer requires a multimodality approach therapy. One of the standard curative treatments is the definitive radiation therapy (RT) associated with androgen deprivation therapy (ADT) [1, 2]. Several randomized trials have shown excellent long-term biochemical outcome with higher radiation doses [3].

Recent literature suggests that adenocarcinoma of the prostate gland is different from many other malignancies, with an average a/b ratio about 1.5 Gy (range, 0.8–2.2). The a/b ratio for late rectal radiation effects is generally calculated between 3 and 4 Gy [4–6]. The rationale of hypofractionated radiotherapy (HFRT) consists in the lower a/b ratio of prostate cancer compared to adjacent organs at risk (OAR). Higher dose per fraction can improve local control increasing the biological effective dose (BED) to the tumor without increasing the risk for late effects. Moreover, using reduced total dose can be unsafe in high-risk patients with Gleason score higher than 7, becauce α/β ratio could not be so low [7].

Hypofractionated radiotherapy can be carried out safety after the development of image-guided radiotherapy (IGRT) and intensity-modulated radiotherapy (IMRT). IMRT has been widely accepted as an efficient technique for dose escalation in localized prostate cancer, and better conformed radiotherapy to pelvic lymph nodes for higher-risk prostate cancer. Although, the pelvic lymph node irradiation is still controversial, randomized data have supported its use [8]. In addition, most of the randomized trials have shown that pelvic irradiation combined long-term ADT had a survival benefit [1, 2].

In 2009, according to these postulations, we started a prospective study at our institution using a hypofractionated schedule with SIB-IMRT-IGRT technique including patients with high-risk prostate cancer. The primary end-point was to evaluate late toxicity and preliminary results.

## Methods

### Patients’ characteristics

Between November 2009 and September 2013, 105 patients with biopsy proven, high-risk prostate cancer were treated with hypofractionated radiation therapy with SIB-IMRT-IGRT. Under a S. Andrea Hospital IRB-approved protocol the data were prospectively collected and retrospectively analyzed to evaluate the efficacy and the tolerance. Written inform consent was obtained by all patients.

A minimum follow up time of 12 months was required as inclusion criteria. For this reason, 82 patients were selected for the statistical analysis in the present study. Median age at diagnosis was 74 years (range 58–88 years). All patients presented cT3/4 N0/N1 M0 clinical stage and/or a Gleason score of ≥ 8 and/or a pre-treatment prostate-specific antigen (PSA) serum level ≥ 20 ng/ml.

Pre-treatment evaluation included: a complete physical examination, PSA level, complete blood counts and standard biochemistry tests, bone scan, total body computed tomography (CT) with contrast medium and prostate magnetic resonance image (MRI) with diffusion and perfusion sequences. Three patients (3,7%) presenting with clinical positive node (external iliac station) were submitted to PET scan with choline to confirm nodal involvement.

Median of PSA value at diagnosis was 9.87 ng/ml (range 3–89.7 ng/ml). Patient characteristics are summarized in Table 1. Written informed consent was provided by all patients.

**Table 1:** **Patients’ characteristics**

***Characteristics***	***Total***	
	***(n = 82)***	
	***n.***	***%***
**Age**		
*<70*	19	23,2
*≥70*	63	76,8
**PSA at diagnosis (ng/mL)**		
*0.1- 10*	42	51,2
*10.1 -19.9*	19	23,2
≥ 20	21	25,6
**Gleason score**		
*<7*	21	25,6
*7*	26	31,7
*>7*	35	42,7
**Clinical stage**		
*T1c*	2	2,4
*T2a*	5	6,1
*T2b*	14	17,1
*T2c*	5	6,1
*T3a*	22	26,8
*T3b*	29	35,4
*T4*	*2*	2,4
*T3bN1*	3	3,7

### Treatment

All patients underwent CT planning (2.5 mm slice thickness) in the supine position with feet rests for the implementation of treatment planning. The preparation for CT scan comprises the administration to a mini enema for rectal emptying and then patients were invited to next urination. Also, they were requested to drink 500 ml of water half an hour before the start of the procedure to fulfil the bladder. Planning CT was fused with MRI images (diffusion ADC map, perfusion series and axial high resolution T2-w) using point-to-point matching to help clinical target volume (CTV) delineation.

Wide description of the treatment patterns was previously reported [9].

Eclipse inverse planning system (Varian) was used to calculate IMRT plans with simultaneous integrated boost. The PTV1 (prostate) was planned to receive 68.75 Gy (2.75 Gy per fraction). The PTV2 (seminal vesicles) and the PTV4 (positive nodes) were planned to receive 55 Gy (2.2 Gy per fraction) and the PTV3 (pelvis) was planned to receive 45 Gy (1.8 Gy per fraction) in 25 fractions. For patients with cT3b, the PTV2 received the same dose as the PTV1. The first 59 patients (72%) received 4 weekly fractions, with overall treatment time of 43 days. The last 23 patients (28%) received 5 weekly fractions, with overall treatment time of 33 days.

Prior to each treatment, patients underwent a Kilo-voltage cone-beam CT that was compared with the planning CT to verify the correct position. The patients’ position was adjusted with an initial automatic bone alignment, followed by a soft tissue alignment using the prostate-rectum interface.

The organ at risk (OAR) dose volume constraints were as follows: for the rectum V52Gy < 35% and V61Gy < 25%; for the bladder V45Gy < 50%; for the femoral head V50Gy < 10%; for peritoneal cavity V54Gy < 5%.

The Normalized Total Dose (NTD) was 82,5 Gy assuming an α/β of 1,5 for the tumour with an iso-effective late complication total dose of 78 Gy delivered in 39 fractions considering an α/β of 3 for rectal late adverse effects.

All patients were submitted to neoadjuvant (starting 3 months before radiation therapy), concomitant and subsequent adjuvant ADT for other 2–3 years using a luteinizing hormone-releasing hormone (LHRH) analogous plus anti-androgen (bicalutamide 50 mg).

From the start of radiation therapy, all patients were advised to follow a low-fibre and low-fat diet and to assume a cranberry based integrator and lactic ferment once daily.

### Follow up

Acute toxicities were recorded using the RTOG/EORTC scale [10] weekly during treatment, at 1 month, at 3 months after treatment completion. All patients underwent weekly clinical evaluation and routine blood examinations during RT. Follow-up was performed every three months for the first two years and every six months up to five years and annually afterwards. Performance status, treatment-related adverse effects, blood count, liver and renal function tests, PSA were assessed at follow-up.

### Statistical analysis

The biochemical failure was defined as the PSA nadir +2 ng/mL according to Phoenix criteria [11]. Overall survival (OS) and disease free survival (DFS) were calculated using the Kaplan**–**Meier method. The chi-square test was performed to compare 4 times weekly and 5 times weekly treatment-related toxicities between these two groups. Statistical analyses were performed using SPSS statistical software package version 13.0 (SPSS, Inc., Chicago, IL). A p value lower than 0.05 was considered as statistically significant.

## Results

The overall median follow-up was 31 months (range 12–58 months). The median follow-up for patients treated 4 times weekly was 40 months (range 13–58 months), whereas for patients treated 5 times weekly was 23,5 months (range 12–31 months).

**Figure 1:**
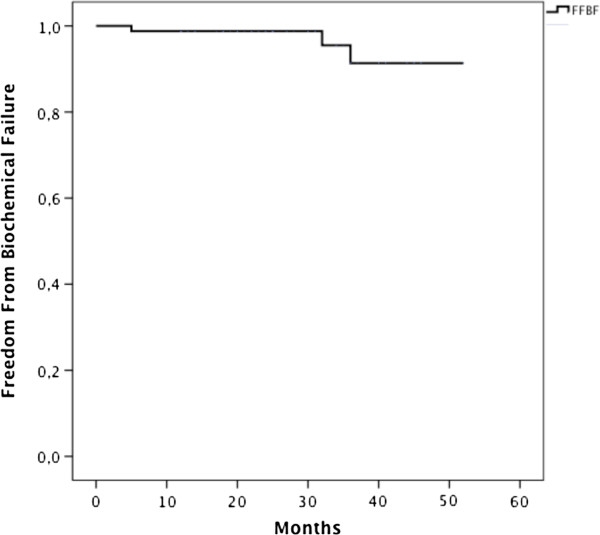
**Freedom From Biochemical Failure (FFBF).**

The median PSA value before radiotherapy was 0,64 ng/ml (range 0.01-12,7 ng/ml), at the first follow-up was 0.08 ng/ml (range 0–4,95 ng/ml) and at the last follow-up was 0.06 ng/ml (range 0–2,9 ng/ml).The actuarial 3-years overall survival (OS) was 98,6%. There was one death at the time of the statistical analysis. The patient died after 13 months from RT completion for cardio-pulmonary disorder and he also presented lumbar-aortic nodal disease progression. The actuarial 3-years freedom from biochemical failure (FFBF) was 91,3% (Figure 1). Three patients developed biochemical failure due to systemic progression: 2 patients had bone metastasis and 1 patient had lumbar-aortic nodes metastasis. None of those patients presented loco-regional relapse.

### Acute toxicities

Toxicities occurred as follows during treatment: grade 1 and grade 2 gastrointestinal (GI) toxicity in 5/82 (6.1%) and 6/82 patients (7.3%), respectively; grade 1, 2 and 3 genitourinary (GU) toxicity in15/83 (18.3%), 3/82 (3.7%) and 1/82 patients (1.2%), respectively.

At one month after treatment completion we observed grade 1 and 2 GI toxicities in 4/82 (4.9%) and in 1/82 patients (1.2%), respectively. Grade 1 and 2 GU toxicities were experienced by12/82 (14.6%) and 2/82 (2.4%) patients, respectively.

At 3 months after radiation treatment grade 1 GI toxicity was observed in 4/82 patients (4.9%). Grade 1 GU toxicity was observed in 8/82 patients (9,8%). No grade ≥ 2 GU and GI toxicities were recorded.

No statistically significant difference was calculated between the two groups treated 4 and 5 times weekly, respectively during the treatment, at 1 and 3 from the end of therapy. Data are summarized in Table 2.

**Table 2:** **Comparison of acute GI and GU toxicities in the 4 W group vs. 5Wgroup**

***Acute toxicity***	***GI***					***GU***				
	***4 W***		***5 W***		p value*	***4 W***		***5 W***		p value*
	***n.***	***%***	***n.***	***%***		***n.***	***%***	***n.***	***%***	
**during RT**										
G1	3/59	5.1	2/23	8.7	0.960	11/59	18,6	4/23	17.4	0.509
≥ G2	4/59	6.8	2/23	8,7		2/59	3.4	2/23	8.7	
**1 months FU**										
G1	2/59	3.4	2/23	8.7	0.291	5/59	8.5	7/23	30,4	0.134
≥ G2	0	0	1/23	4,3		1/59	1.7	1/23	4.3	
**3 months FU**										
G1	3/59	5,1	1/23	4,3	0,692	4/59	6,8	4/23	17,4	0,320
≥ G2	0	0	0	0		0	0	0	0	
**6 months FU**										
G1	1/59	1,7	1/23	4,3	0,632	4/59	6,8	2/23	8,7	0,965
≥ G2	0	0	0	0		0	0	0	0	
**Last FU**										
G1	1/59	1,7	0	0	0,582	1/59	1,7	1/23	4,3	0,642
≥ G2	0	0	0	0		0	0	0	0	

*Chi-square test.

### Late toxicities

At 6 months from the end of therapy, 2 patients (2.4%) presented grade 1 GI toxicity and 6 patients (7.3%) presented grade 1 GU toxicity. No grade ≥ 2 toxicities were recorded.

At the last follow-up, grade 1 GI and GU toxicities were observed in 1 patient (1.2%) and 2 patients (2.4%), respectively. Only 1 patient (1.2%) developed grade 2 GI toxicity No grade ≥ 3 toxicities were recorded.

No statistically significant difference was recorded between two groups treated 4 and 5 times weekly, respectively. Data are summarized in Table 2.

## Discussion

The lower a/b ratio of prostate cancer compared to the adjacent OAR predicts for the sparing of normal tissues when hypofractionated RT is carried out [12]. In addition, IMRT and daily image guidance have been shown to improve biochemical disease free survival (bDFS) and reduce toxicity [13]. The results of the present prospective trial have shown that a concomitant SIB-IMRT- IGRT and long-term ADT are safe and well tolerated with low rates of toxicity.

Moreover, we reported low rates of acute toxicity during the treatment with grade 1 and grade 2 GI toxicity of 6.1% and 7.3%, respectively, and grade 1, 2 and 3 GU toxicity of 18.3%, 3.7% and 1.2%, respectively. At 3 months after radiation treatment only 4.9% presented grade 1 GI toxicity and 9.8% presented grade 1 GU toxicity. In the current study, we found that the overall treatment time (4 or 5 times weekly) had no impact on acute toxicity rates.

The present study reports outcome regarding biochemical control and late toxicity after a median follow-up of 31 months. The actuarial 3-years OS was 98.6%. The actuarial 3-years DFS was 91.3% (3 patients developed biochemical failure due to systemic progression: 2 patients had bone metastasis and 1 patient had lumbar-aortic nodes metastasis). None of those patients presented loco-regional relapse.

In the literature, there are several randomized controlled trials with mature follow-up addressing moderate hypofractionated radiotherapy in clinically localized prostate cancer. Arcangeli et al. [14] reported the Regina Elena study, which randomized 168 patients to 80 Gy in 2-Gy fractions over 8 weeks versus 62 Gy in 3.1-Gy fractions over 4 weeks. All patients received 9 months of neoadjuvant, concurrent, and adjuvant androgen deprivation therapy. They demonstrated superior 3-year freedom from biochemical failure in the hypofractionated arm (FFBF 87% vs. 79%) after a median follow-up time of 3 years. In a recently update of the previous study, no significant difference was found in terms of FFBF, overall survival, or cause-specific survival between treatment arms, after a median follow-up of 70 months; there were also no difference in late toxicity [15]. The Fox Chase trial [16] randomized 303 men with intermediate- and high-risk prostate cancer to 76 Gy in 2-Gy fractions over 7.5 weeks versus 70.2 Gy in 2.7-Gy fractions over approximately 5 weeks, using IMRT. Median follow-up was 68.4 months. The 5-year rates of biochemical and/or clinical disease failure (BCDF) were 21.4% (95% CI, 14.8% to 28.7%) for conventional IMRT and 23.3% (95% CI, 16.4% to 31.0%) for hypofractionated IMRT. There were no statistically significant difference in late toxicity between the arms; however, in subgroup analysis, patients with compromised urinary function before enrolment had significantly worse urinary function after hypofractionated IMRT. The MD Anderson [17] randomized 204 men to 75.6 Gy in 1.8-Gy fractions over 8.5 weeks versus 72 Gy in 2.4-Gy fractions over 6 weeks. After a median follow-up of 4.7 years, no intergroup difference in 5-year FFBF (hypofractionated vs. conventional, 96% vs. 92%) has been demonstrated. There was no significant difference in toxicity, although the hypofractionated arm trended toward worse GI toxicity. Three large, multi-institutional, phase III non-inferiority studies from the MRC, RTOG, and OCOG would provide better evidence on moderate hypofractionation over the next few years.

Limited data from prospective studies reported late toxicity dose-escalation with hypofractionated RT on prostate cancer patients after longer follow-up. These studies treated the prostate, the seminal vesicles, with or without the pelvic lymph nodes.

Kupelian et al. [18] reported 3.1% of grade 2, 1.3% of grade 3, and 0.1% of grade 4 late toxicity rates after a median 45 months of follow-up in 770 patients treated with IMRT hypofractionated schedule of 70 Gy/2.5 Gy in 28 fractions to the prostate, including or not seminal vesicles. In particular, the actuarial 5 years rates of grade ≥ 2 rectal late toxicity was 6% and grade ≥ 2 late urinary toxicity at 5 years was 7%. However, none of those patients received whole pelvic RT, and only 60% had adjuvant androgen deprivation. The role of elective radiotherapy on the pelvic nodal regions in clinically node-negative patients remains controversial [19, 20]. The RTOG 9413 [21] trial is a four-arm study of whole-pelvis RT versus prostate-only RT associated with hormonal therapy. Patients who received whole-pelvis RT + ADT experienced grade ≥ 2 late GI and GU toxicity at 5 years of 15.2% and 14.9%, respectively. In our study, the rates of moderate and severe toxicity were lower compared to the previous studies.

Intensity-modulated radiation therapy is emerging as an important technique for more conformal targets of pelvic lymph nodes for higher-risk prostate cancer treatment [10]. Limited clinical data exist on this topic. The current data suggest a reduced toxicity profile compared to conventional and 3D whole pelvic techniques [22, 23]. In a study by Zelefsky et al. [24], the 3-year actuarial incidence of late Grade ≥ 2 GI toxicity in patients treated up to 81 Gy with IMRT was 2% compared with 14% in those treated with 3D-CRT and the same dose. The 3-year bNED rate was similar in patients treated with both techniques and varied from 92% for favourable and 81% for unfavourable risk groups. McCammon et al. [25] retrospectively analysed patients underwent pelvic IMRT (50.4 Gy at 1.8 Gy/fraction) and concomitant boost (70 Gy at 2.5 Gy/fraction) + ADT. Late Grade 2, grade 3 and grade 4 GI toxicities (proctitis) were observed in 6.7%, 3.3%, and 3.3%, respectively, after 24 months of median follow-up. Late grade 2 GU toxicity (urinary frequency/urgency) was observed in 10% of the patients; no grade ≥ 3 was reported.

In the present study the overall median follow up was 31 months. At 6 months after the end of RT 2.4% of the patients presented grade 1 GI toxicity and 7.3% grade 1 GU toxicity. No grade ≥ 2 toxicity was recorded. At last follow-up 1.2% of the patients presented grade 1 GI toxicity, 1.2% of the patients had grade 2 GI toxicity, and 2.4% of the patients had grade with 1 GU toxicity. No grade ≥ 3 toxicity was recorded. No significant difference was recorded between the two groups treated 4 times weekly and 5 times weekly. Compared to our study, Quon et al. [26] reported higher rates of acute and late toxicities in patients with high-risk prostate cancer treated using IMRT-SIB without IGRT and a total dose of 45 Gy (1.8 Gy/fraction) to the pelvic lymph nodes + concomitant boost of 22.5 Gy (2.7 Gy/fraction) to the prostate. A recent study by Zelefsky et al. [27] reported improved biochemical relapse-free survival in patients treated with daily IGRT compared to patients treated without IGRT. The lower toxicity rates achieved in our study are likely a result of the advantage of IMRT combined to IGRT.

In our study, we treated 59 patients with four times weekly radiotherapy considering the hypothesis of Fowler et al. [28] to maintain BED10 value ≤ 60 Gy. This value was calculated using the time-adjusted BED formula .

Based on simple linear quadratic (LQ) model including cell proliferation [29]. Specifically, the overall treatment time was represented by *T*, with the first day designated Day 0. The parameters were selected with a comprehensive review of clinical results from several dozen published schedules used for head and neck cancer treatment [30]. They included α/β = 10 Gy; α = 0.35 Gy^-1^; an onset time for repopulation in human oral mucosa of *T*_*k*_ = 7 days [31]; and an average doubling time of *T*_*p*_ = 2.5 days thereafter. Although this formula is likely simple containing only two repopulation rates, zero and T_p_, it has served well in comparing tumor BEDs in the head and neck schedules studied [5].

The purpose of this schedule is to moderately reduce the overall treatment time, in order to maintain acceptable acute toxicity rates. Based on the low rate of toxicity reported in the first 59 patients we decided to reduce the total treatment time up to 33 days and, then, the last 23 patients received five fractions per week, with no calculated difference in terms of observed toxicity rates between the two groups on the statistical analysis. Regard to the postulation of Fowler the reported low rate of toxicity also in patients treated in 33 days may be explained by the use of IMRT combined to IGRT and reduced margin (5 mm) from CTV to PTV. Our study also presented limits as the relatively short follow-up time.

## Conclusions

Our study demonstrated that hypofractionated radiotherapy using modern technique is efficient and safe. Good outcome in terms of survival and low toxicity rates were observed. The reduction of overall treatment time does no increase toxicity rates. Longer follow-up is necessary to confirm these data. Further studies with longer follow-up are needed to better define outcomes and late toxicity rates after hypofractionated radiotherapy in high-risk prostate cancer patients.
